# Anemia increases the mortality risk in patients with stroke: A meta-analysis of cohort studies

**DOI:** 10.1038/srep26636

**Published:** 2016-05-23

**Authors:** Zhanzhan Li, Tao Zhou, Yanyan Li, Peng Chen, Lizhang Chen

**Affiliations:** 1 Department of Oncology, Xiangya Hospital, Central South University, Changsha, Hunan Province 410008, China; 2 The Third Affiliated Hospital of Southern Medical University, Guangzhou, Guandong Province 510000, China; 3 Department of orthopedic, Xiangya Hospital, Central-south University, Changsha, Hunan Province 410008, China; 4 Department of Epidemiology and Health Statistics, School of public health, Central South University, Changsha, Hunan Province 410078, China

## Abstract

The impact of anemia on the outcome of patients with stroke remains inconsistent. We performed a meta-analysis of cohort studies to assess the mortality risk in stroke patients with and without anemia. Systematic searches were conducted in the PubMed, China National Knowledge Infrastructure, Web of Science and Wanfang databases to identify relevant studies from inception to November 2015. The estimated odds ratio with a 95% confidence interval was pooled. subgroup analyses and sensitivity analyses were also conducted. We used Begg’s funnel plot and Egger’s test to detect the potential publication bias. Thirteen cohort studies with a total of 19239 patients with stroke were included in this meta-analysis. The heterogeneity among studies was slight (*I*^*2*^ = 59.0%, *P* = 0.031). The results from a random-effect model suggest that anemia is associated with an increased mortality risk in patients with stroke (adjusted odds ratio = 1.39, 95% confidence interval: 1.22–1.58, *P* < 0.001). The subgroup analyses are consistent with the total results. This meta-analysis of 13 cohort studies finds that anemia increases the mortality risk in patients with stroke. Future studies should perform longer follow-up to confirm this finding and explore its possible mechanism.

Anemia is a common condition for the older population, and its prevalence increases with age. Previous studies have found that anemia is associated with mortality, poor physical performance and disability^1^,^2^,^3^. Additionally, anemia is an independent predictor of in-hospital mortality both in patients with acute myocardial infarction and in patients with congestive heart failure^4^,^5^. The cerebral oxygen supply depends on the cerebral blood flow and arterial oxygen content, and the hemoglobin level determines the arterial oxygen content^6^. Anemia may cause a reduction in the oxygen-carrying ability, alterations in blood viscosity and impaired cerebral regulation^7^. It is reasonable that low hemoglobin or anemia has disadvantageous influences on the brain. Unfortunately, such a situation is adverse for patients with stroke, and the current treatment guidelines do not recommend an appropriate cutoff of hemoglobin level for stroke^8^.

Stroke has many well-known risk factors, such as diabetes mellitus, smoking, aging, dyslipidemia and hypertension^9^,^10^. Recent studies have suggested that anemia is a risk factor for ischemic stroke, and is related to high mortality in the hospital. However, the impact of anemia on the mortality risk of stroke remains unclear, and several epidemiological studies show inconsistent results^11^,^12^,^13^. To our knowledge, the consistency and quality of evidence on this topic have not been well reviewed, which limits our comprehensive understanding of the impact of anemia on the outcomes and prognosis of stroke. Based on the current findings and accumulating evidence, we conducted a meta-analysis of published cohort studies to assess the mortality risk in patients with stroke.

## Results

### Study flow and characteristics

**Figure 1:**
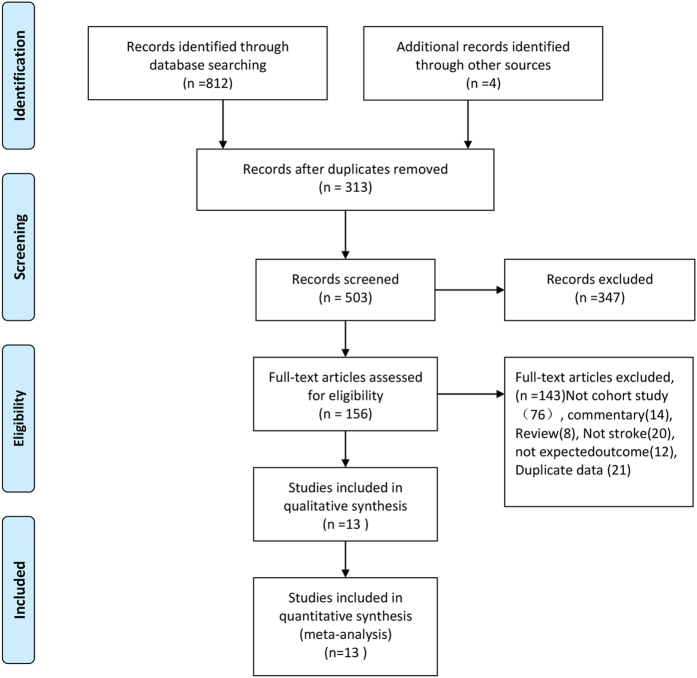
The process of study selection.

Our initial search returned 813 records and three additional records were identified through retrieving references lists. Of these, 503 records were left for further screening after the duplicates were removed. A total of 347 records were excluded for a variety of reasons, such as being a review, commentary, case reports, duplicates, letter, or irrelevant studies. A total of 156 records were ready for the third stage, and 143 records were excluded after further screening. Finally, 13 were cohort studies entered into our meta-analysis^12^,^13^,^14^,^15^,^16^,^17^,^18^,^19^,^20^,^21^,^22^,^23^,^24^. The specific process of study selection is presented in Fig. 1.

Table 1 shows the general characteristics of the 13 cohort studies with a total of 10009 participants included in the meta-analysis. The included studies were published from 2007 to 2015. Seven studies were from China^12^,^15^,^16^,^18^,^19^,^20^,^23^, and the remaining six ones were from the USA^22^, Denmark^13^, Greece^14^, India^17^ Israel^21^, and Canada^24^. Two studies had a retrospective study design and 10 had a prospective cohort study design. The sample sizes ranged from 66 to 9230^14^,^24^. The maximum duration of follow-up was three years^12^ and the minimum was 48 hours^17^. The prevalence of anemia ranged from 0.11% to 39.4%. Seven studies were conducted in a population with acute stroke, and 6 were conducted in patients with non-acute stroke. Six studies provided the information from initial symptoms to admission (within 7 days), the rest are lack of this information. Of these,13 studies treated death as the measure index, and two studies also include disability. Ten studies adopted the WHO criteria (male:<130 g/L, female:<120 g/L), 2 studies used self-defined criteria (male:<140 g/L, female:<120 g/L and male, and female:<100 g/L), and 1 study adopted the China criteria (male<120 g/L, and female:<110 g/L). The mean NOS score of study quality was 7.1. The additional file 1 shows the quality assessment of the study according to the NOS criteria.

### Anemia and the mortality risk of stroke

**Figure 2:**
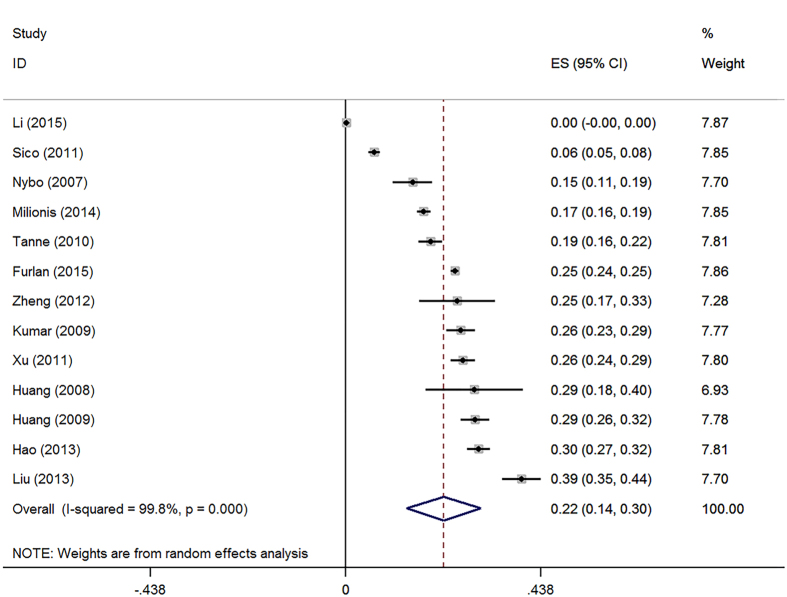
Prevalence of anemia in patients with stroke.

**Figure 3:**
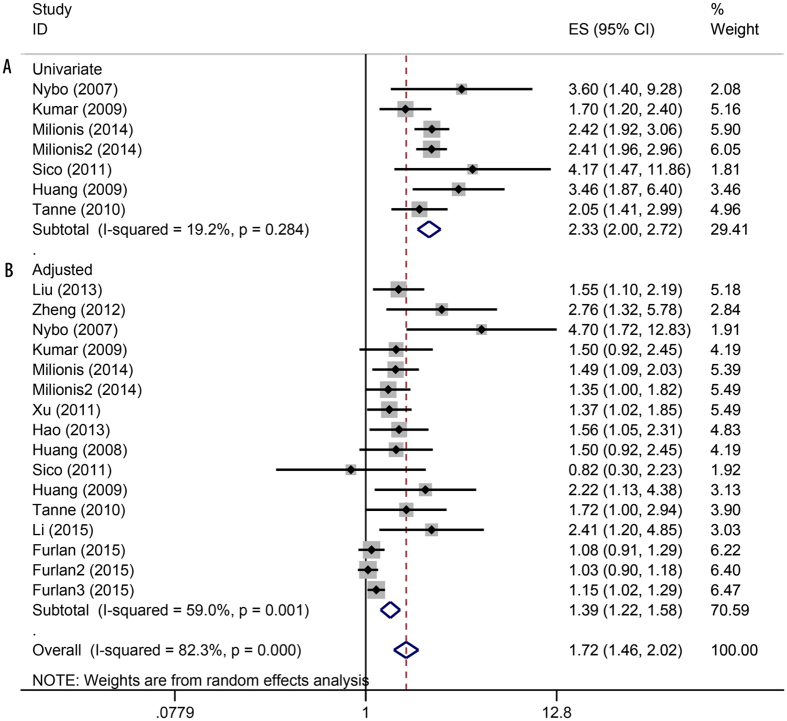
Forest plot showing the mortality risk of anemia in patients with stroke.

Figure 2 shows the pooled prevalence of anemia in patients with stroke. The results from 13 studies show that the prevalence was 21.9% (95%CI: 13.6–30.3%). Figure 3 shows the estimated OR of anemia for stroke. The univariate results include six studies and the adjusted results have 13 studies. A total of 19,239 patients are included in the meta-analysis. The heterogeneity within the study is low (I^2^ = 0% to 59%). The random-effects model was conducted for the pooled OR. Both the univariate and adjusted analyses found that anemia is associated with an increased mortality risk in patients with stroke (for univariate: OR = 2.33, 95%CI: 2.00–2.72; for adjusted: OR = 1.39, 95%CI: 1.22–1.58). Table 2 also shows the subgroup results from the region and type of disease. The results of the subgroup analyses are consistent with the adjusted pooled results, and the anemia remains related to mortality of stroke (Table 2).

### Sensitivity analysis

We first assessed the influence of each study on the increased mortality risk by omitting one study each time. The results found no extensive change for the pooled estimation. To further examine the stability of the results, we also conducted extra analyses for the following four types: Type I: excluding 2 retrospective studies (OR = 1.36, 95%CI: 1.20–1.55); Type II: excluding 1 study with small sample size (OR = 1.58, 95%CI: 1.37–1.82); Type III: excluding 2 studies with low prevalence of anemia (OR = 1.50, 95%CI: 1.33–1.70); Type IV: excluding 3 studies with less than 3 months of follow-up (OR = 1.64, 95%CI: 1.41–1.91); Type V: excluding 3 studies with self-defined criteria of anemia (OR = 1.57, 95%CI: 1.38–1.78). The analyses suggested that our results are sufficiently robust to identify the increased mortality risk of anemia for patients with stroke.

### Publication bias

**Figure 4:**
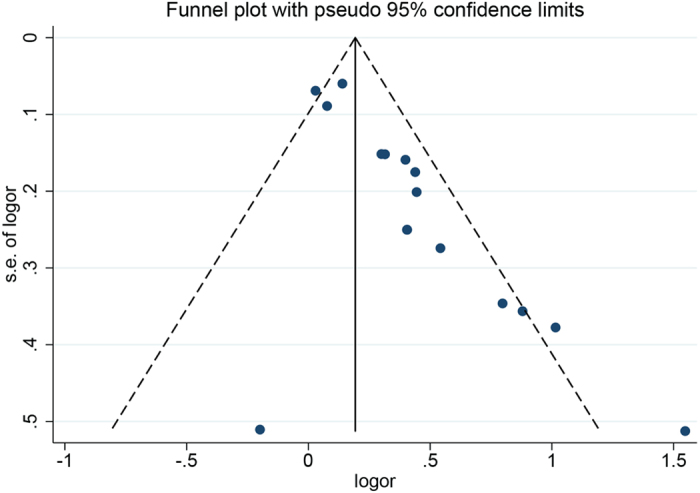
Funnel plot for the mortality risk of anemia in patients with stroke.

The Begg’s and Egger’s test indicated that a slight publication bias exists (*P* < 0.05). Asymmetry is also found in the medium part of the funnel plot, in which approximately two negative studies are missing (Fig. 4). This asymmetry may potentially overestimate the risk of anemia for patients with stroke. Seven of the included studies are from China, which this may be the reason for this finding.

## Discussion

The present meta-analysis of 13 cohort studies shows that anemia is an independent risk factor of unfavourable outcomes in patients with stroke. The stroke patients with anemia had a 39% increase in mortality risk compared with those without anemia. In addition, this relationship was found in some subgroup analyses. Our findings suggest that early intervention for patients with stroke and anemia could be important to decrease the risk of adverse clinical outcomes.

Previous studies have confirmed the relationship between anemia and short-term adverse outcomes in other patients settings such as chronic heart failure^25^, type 2 diabetes patients with chronic kidney disease^26^ and myocardial infarction^27^. The present study provides further support for the relationship between anemia and vascular condition. The prevalence of anemia in acute patients range from 17% to 29%^11^,^12^. We also estimated the prevalence of anemia of stroke patients in this meta-analysis. Our results found that the pooled prevalence of anemia in patients with stroke was 21.9% (95%CI: 13.6–30.3), which was significantly higher than the reported prevalence in the general elderly population, in which the prevalence was 7%^28^. Of the 13 studies, there were inconsistent results for the association between the mortality risk of stroke and anemia. In contrast to these reports, 2 studies found that anemia was not always associated with mortality in patents with stroke. Sico *et al.* conducted a 1-year follow-up in less severe ischemic stroke patients with a sample size of 1306. They found that anemia might be independently associated with outcome in patients with less severe stroke (adjusted OR = 4.17; 95% CI: 1.47–11.90), but not in patients with more severe strokes (adjusted OR, 0.82; 95%CI: 0.30–2.22). In parallel with these findings, Hao *et al.* found that anemia was not a risk factor of death and disability outcome (adjusted OR = 1.01, 95%CI: 0.71–1.44) in patient with acute ischemic stroke^16^. The reason for this result could be that the severity of stroke contributed to the mortality risk more than the other existing complications such as the presence of anemia^17^. Interestingly, Sico *et al.* also suggested a J-shaped relationship between the hematocrit level and a poor prognosis in severe stroke patients. This only explains why patients with more severe stroke tend to have adverse outcomes but did not confirm the relationship and risk in patients with less severe stroke. The remaining studies reported an increase risk of mortality in patients with anemia.

The oxygen supply of the brain is closely related to the hemoglobin level and blood viscosity. There could be three stages during the process of the adverse impact on the outcomes in patients with stroke. First, early-stage injury may occur to cerebral regulation when hemoglobin is at a low level. During this period, the cerebral vascular system can continue to regulate itself for normal function. A lower hemoglobin level is associated with a higher relative cerebral blood flow within the middle/inferior frontal regions, whereas higher hemoglobin level is associated with lower parenchymal cerebral blood flow and a decrease in parenchymal cerebral blood flow over time^29^,^30^. When anemia occurs in patients with stroke, the lack of oxygen and energy supply will have a great significant influence on the cerebral vascular regulation over time. In addition to other medical complications, anemia dominates for the accumulation damage course of cerebral autoregulation. With the progress of the disease, patients with anemia will suffer from many complications, such as heart failure^31^, chronic kidney disease^32^ and vascular endothelial cells^33^,^34^, which play a more important role than anemia in the development of stroke. This effect may partly explain why Sico and colleagues found a positive relationship in patients with less severe stroke but not in severe stroke^17^. In addition, anemia may be suggested to be related to the inflammation response, and some inflammatory markers are increased in patients with anemia, such as C-reactive protein, tumour necrosis factor-α (TNF-α), and some interleukins (ILs)^35^. These inflammatory markers could affect the prognosis after stroke. TNF-α is involved in the process of ischemic injury, and increased IL-6 has been found in the adverse prognosis after stroke. Moreover, C-reactive protein may be suggested to be related to increased mortality risk after stoke^36^,^37^,^38^, and anemia may affect outcomes after stroke through its relationship with inflammation.

The strengths of our meta-analysis are that only cohort studies with high quality were included, and the adjusted results were reported. In addition, we conducted study selection, data extraction and analyses, which strictly complied with the MOOSE (Meta-analysis of Observational Studies in Epidemiology Statement) guidelines. These could reduce the possibility of recall bias, which is particularly important for observational studies.

### Study limitations

There are several limitations for our meta-analysis. First, subgroup analyses are not the same as the adjusted pooled results with controlling for major potential confounding factors. We cannot conduct combined analysis because of the lack of data in subgroup analyses. There may be some confounders affecting the results, such as hemoglobin measurement, which occurs by capillary, venous, POCT equipment. Despite the report of a positive relationship between anemia and mortality risk of stroke, a study by Tnne *et al.* included some patients with intracerebral hemorrhage and a high loss of follow-up^21^. Second, patients with anemia could have more poor health problems, which could result in increasing the risk of mortality. Third, the maximum follow-up time was 3 years, and therefore assessment of long-term effects of anemia post stroke is lacking. Some studies have suggested a significant association between anemia and adverse outcomes in patents with stroke, although the relationship is weak. The cut-off values of 95%CI are approximately equal to one. Anemia is more likely a bystander effect. A study with a longer follow-up time is needed. Finally, the funnel plot indicated the presence of publication bias, which may overestimate the risk effect of anemia because some negative results are missing.

In conclusion, this meta-analysis of 13 cohort studies finds that anemia increases the mortality risk in patients with stroke. Future studies should conduct longer follow-up to confirm and explore the possible mechanism for this finding.

## Methods and Materials

The ethical approval is not necessary for the present study because this is a meta-analysis of the published studies. We conducted the meta-analysis and systematic review in accordance with the guidelines of MOOSE^39^.

### Literature Search Strategy

We conducted systematic searches in the PubMed, China National Knowledge Infrastructure (CNKI), Web of science and Wanfang databases to identify relevant studies without language restrictions from inception to November 2015. The search terms included ‘anemia’, ‘anaemia’, ‘hypohemia’, ‘stroke’, ‘acute ischemic stroke’, ‘ischemic stroke’, ‘hemorrhagic stroke’, ‘AHS’, and ‘apoplexy’. We also retrieved the reference lists from recent reviews to obtain other potentially eligible studies that were not identified in our initial search.

### Criteria for Inclusion

A study must have met the following criteria: (1) type of study: a prospective or retrospective study; (2) study population: patients diagnosed with stroke, including acute or non-acute ischemic stroke, hemorrhagic stroke; (3) comparison of factor: with and without anemia; (4) outcome: the primary outcome is death or with disability; (5) Definition: studies give specific WHO or other definition of anemia. (6) information: studies at least provide the risk estimation of anemia in a multivariate analysis. In addition, we only included those studies that contained the most recent and complete information if duplicated data were found in several studies.

### Data Extraction and Quality Assessment

We used a standardized data collection sheet to extract the following information: first author, year of publication, region, study design, age of the study population, duration of follow-up, sample size, prevalence of anemia, number of female and male, adjusted factors in the multiple factor analysis, outcome measurement, the univariate estimated odds ratio (ORs) with 95% confidence intervals (95%CIs) and the adjusted ORs with 95%CIs. We also contacted the corresponding author of articles to obtain more information.

We used NOS (Newcastle-Ottawa Scales) items to assess the quality of included studies^40^. The NOS includes selection, comparability and outcome. The selection item includes four sub-item: exposed cohort, nonexposed cohort, ascertainment of exposure and outcome of interest. The outcome includes assessment, length of follow-up and adequacy of follow-up. We evaluated the quality of included studies as the following rules: priority (7–8 adequate items), high quality (equal or more than 5 items), low quality (less than 5 items) and extreme low quality (no description of study methods).

Two investigators independently performed the selection of included studies, data collection and quality assessment. A third investigator solved any disagreements.

### Statistical Analysis

Nearly all studies used OR as a risk estimation between anemia and different types of stroke. Therefore, we pooled the OR of the included studies in this meta-analysis. We first estimated the prevalence of anemia in patients with stroke using DerSimonian-Laird in random effects model. The Cochran Q test and I^2^ were applied to examine the heterogeneity within studies. For the I^2^ statistic, low level was less than 25%, moderate was 25–50%, and high level was more than 50%^41^,^42^. Random effects models are used for significant heterogeneity; otherwise fixed effects models are applied. To explore the possible inconsistency of results, we also conducted subgroup analyses in univariate and multivariate analyses, type of disease (acute vs non-acute), region (China vs others), follow-up duration (<6 months vs ≥6 months), and male/female ratio (median: ≤1.2 vs >1.2). To assess the stability of the pooled results, we conducted 5 types of sensitivity analyses: Type I, excluding 2 retrospective studies; Type II, excluding 1 study with small sample size; Type III, excluding two studies with low prevalence of anemia; Type IV, excluding 3 studies with less than 3 months of follow-up; Type V, excluding 3 studies with self-defined criteria of anemia. We also evaluated the influence of each study on the summarized estimation by omitting on study in each time. We used Begg’s funnel plot and Egger’s rank correlation test to detect the potential publication bias^43^,^44^. *P* < 0.05 was considered statistically significance. All statistical analyses were conducted using the Stata 12.0 software (Stata Corporation, College Station, TX, USA).

## Additional Information

**How to cite this article**: Li, Z. *et al.* Anemia increases the mortality risk in patients with stroke: A meta-analysis of cohort studies. *Sci. Rep.* **6**, 26636; doi: 10.1038/srep26636 (2016).

## Supplementary Material

Supplementary Information

## Figures and Tables

**Table 1:** General characteristics of 13 cohort studies included in the meta-analysis.

First Author	Region	Study design	Age	Follow-up (rate of loss follow-up)	Patients	Sample size	Time from initial symptoms to admission	Anemia (%)	Male/female	Adjustments	Outcome	Criteria for Anemia (Hb)
Liu 2013	China	Prospective Cohort study	51–87	6 months (4.6%)	Ischemic stroke	481	–	39.40	216/243 (0.89)	age, sex, medical treatment, glucose, smoke, hypertension, CAD, history of cardiovascular disease, infection	Death	Male:<130 g/L, female:<120 g/L
Zheng 2012	China	Retrospective cohort study	32–91	3 months (0.0%)	hemorrhagic stroke	101	–	0.25	65/36 (1.81)	age, gender, SBP, infection, low hemoglobin, recognition,	Death	Male:<130 g/L, female:<120 g/L
Nybo 2007	Denmark	Prospective Cohort study	Anemia:73 Non Anemia: 68	6 months (0.0%)	Ischemic Stroke	250	≥7 days	15.00	133/117 (1.14)	Age, gender, SSS<30, heart failure, and or renal function impaired	Death	Male:<130 g/L, female:<120 g/L
Kumar 2009	USA	Prospective Cohort study	71.3(mean)	1 month (1.3%)	Acute hemorrhage stroke	685	–	25.80	278/307 (0.91)	age, sex, IVH, ICH volume, glucose, WBC, SBP, DBP, warfarin	Death	Male:<130 g/L, female:<120 g/L
Milionis 2014	Greece	Prospective Cohort study	Anemia:73.45 Non anemia: 68.82 ± 25.68	1 years (0.0%)	acute ischemic stroke	2439	–	17.50	1347/1065 (1.26)	age, sex, hemoglobin, C-reaction protein, CAD, NIHSS,	Death	Male:<130 g/L, female:<120 g/L
Xu 2011	China	Prospective Cohort study	68.6 ± 12.2	1 month (0.0%)	acute stroke	1014	–	26.30	559/455 (1.23)	vascular risk factors, cardiovascular disease, proteinuria, sex, age	Death disability	Male:<130 g/L, female:<120 g/L
Hao 2013	China	Prospective Cohort study	Anemia:68.28 Non anemia: 64.35	1 year (0.0%)	Ischemic stroke	1176	<24 hours	29.80	674/502 (1.34)	age, sex, NIHSS, vascular risk factors renal function	Death disability	Male:<130 g/L, female:<120 g/L
Huang 2008	China	Prospective Cohort study	67.6	2 years (9.7%)	Ischemic stroke	66	<24 hours	28.80	51/15 (3.4)	Age, sex, presence of IHV, ICH volume, warfarin, glucose, WBC	Death	Male:<120 g/L, female:<110 g/L
Sico 2011	India	Retrospective cohort study	71.5	48 hours (0.0%)	ischemic stroke	1306	<2 days	6.40	751/555 (1.35)	age, sex, race, medical history, Charlson comorbidity index, score, hematocrit,	Death	Male and female:<100 g/L
Huang 2009	China	Prospective Cohort study	Anemia:71.6 No anemia: 6 67.4	3 year (4.9%)	Ischemic Stroke	774	<2 days	29.00	412/362 (1.14)	age, sex, CKD, CAD, hyperkinemia	Death	Male:<130 g/L, female:<120 g/L
Tanne 2010	Israel	Prospective Cohort study	70.6	1 year (0.0%)	Acute stroke	859	–	19.00	497/362 (1.37)	age, sex, stroke type, stroke severity, prior disability, CKD, other cardiac disease and malignancy	Death disability	Male:<130 g/L, female:<120 g/L
Li 2015	China	Prospective Cohort study	Anemia:68.56 Non anemia: 63.49	3 months (0.0%)	acute stroke	858	<24 hours	0.11	547/311 (1.76)	age, sex, NIHSS, cardiovascular risk factors, alcohol, tumor	Death	Male:<130 g/L, female:<120 g/L
Furlan 2015	Canada	Prospective Cohort study	–	7days, 1, 3 months	acute stroke	9230	–	24.5%	4747/4483(1.06)	age, sex, ethnicity, alcohol, cardiovascular risk factors, smoking, history of disease	Death	Male:<140 g/L, female:<120 g/L

*CAD: coronary heart disease; SBP: systolic blood pressure; DBP: diastolic blood pressure; IVH: intraventricular hemorrhage; ICH:intracranial hemorrhage; CKD: chronic kidney disease; NIHSS: national institutes of health stroke scale; Hb: hemoglobin.

**Table 2:** Pooled prevalence and combined odd ratio of anemia for stroke.

Category	Subgroup	NO. of Studies	Pooled OR/prevalence	95%CI(%)	*Z*	*P*	*I*^*2*^(%)	*P_*hetero*_
Prevalence of anemia		13	21.9%	13.6–30.3	5.17	0.000	99.8	0.000
Total								
	univariate	6	2.33	2.00–2.72	10.76	0.000	19.2	0.284
	Adjusted	13	1.39	1.22–1.58	4.94	0.000	59.0	0.031
Region								
	China	7	1.60	1.36–1.89	5.59	0.000	0.0	0.486
	Others	6	1.23	1.07–1.41	2.97	0.003	59.0	0.021
Type of disease								
	Acute	7	1.28	1.13–1.46	3.75	0.000	0.0	0.014
	Non-acute	6	1.66	1.27–2.17	3.72	0.000	29.7	0.212
Follow-up duration	<6 months	6	1.244	1.08–1.43	3.07	0.000	56.2	0.019
	≥6months	7	1.58	1.34–1.86	5.42	0.000	10.7	0.348
Male/Female ratio	≤1.2	5	1.26	1.06–1.49	2.68	0.007	67.0	0.026
	>1.2	8	1.50	1.30–1.72	5.70	0.000	0.0	0.000
Sensitivity analysis								
	Type I*	11	1.36	1.20–1.55	4.78	0.000	58.4	0.003
	Type II*	12	1.58	1.37–1.82	6.30	0.000	14.7	0.300
	Type III*	11	1.50	1.33–1.70	6.37	0.000	0.0	0.550
	Type IV*	10	1.64	1.41–1.91	6.29	0.000	13.0	0.323
	Type V*	10	1.57	1.38–1.78	6.97	0.000	11.6	0.334

*P_hetero_ for heterogeneity.

*Type I, excluding two retrospective studies; Type II, excluding one studies with small sample size; Type III, excluding two studies with low prevalence of anemia; Type IV, excluding three studies with less than three months of follow-up. Type V, excluding three studies with self-defined criteria of anemia.
