# Supplementary material for: Anemia increases the mortality risk in patients with stroke: A meta-analysis of cohort studies
Source: Sci Rep. 2016 May 23;6:26636. doi: 10.1038/srep26636 (PMC4876389; doi:10.1038/srep26636)
Supplement: Supplementary Information [file srep26636-s1.pdf]

**Title:** Anemia increases the mortality risk in patients with stroke: A meta-analysis of cohort studies

Zhanzhan Li, Tao Zhou Yanyan Li, Peng Chen, Lizhang Chen

**Additional file 1** Methodological quality assessment (risk of bias) of included studies by Newcastle-Ottawa Scales

| **Study** | **Selection** | | | | **Comparability** | **Outcome** | | | **Total score** |
|---|---|---|---|---|---|---|---|---|---|
| | Exposed Cohort | Nonexposed Cohort | Ascertainment of exposure | Outcome of interest | | Assessment of outcome | Length of follow-up | Adequacy of follow-up | |
| Liu, 2013 | * | * | * | * | ** | * | - | - | 7 |
| Zheng,2012 | * | * | * | * | * | * | - | - | 6 |
| Nybo,2007 | * | * | * | * | ** | * | - | - | 7 |
| Kumar,2007 | * | * | * | * | ** | * | - | - | 7 |
| Milionis,2009 | * | * | * | * | ** | * | * | - | 8 |
| Xu,20111 | * | * | * | * | * | * | - | - | 6 |
| Hao,2013 | * | * | * | * | ** | * | * | - | 8 |
| Huang,2008 | * | * | * | * | * | * | * | - | 7 |
| Sico,2011 | * | * | * | * | ** | * | - | - | 7 |
| Huang,2009 | * | * | * | * | ** | * | * | - | 8 |
| Tanne,2010 | * | * | * | * | ** | * | - | - | 7 |
| Li,2015 | * | * | * | - | ** | * | - | - | 6 |
| Furlan,2015 | * | * | * | * | ** | * | * | - | 8 |
